# Supplementary material for: Bacterial persistence is essential for susceptible cell survival in indirect resistance, mainly for lower cell densities
Source: PLoS One. 2021 Sep 2;16(9):e0246500. doi: 10.1371/journal.pone.0246500 (PMC8412311; doi:10.1371/journal.pone.0246500)
Supplement: S2 Fig — Results of simulations when we assumed that the persister population decays according to an exponential and that persister cells leave the dormant state as soon as the medium becomes detoxified. (DOCX) [file pone.0246500.s002.docx]

**S2 Figure - Persister and non-persister cells and their respective descendants through time considering τ_0_ = 50,** $\boldsymbol{k}_{\boldsymbol{1}}$ **= 0.055,** $\boldsymbol{k}_{\boldsymbol{2}}$ **= 0.01.** The horizontal axis, representing time (minutes), is linear, and the vertical axis is on a logarithmic scale and represents the susceptible cells density. The blue circles represent the theoretical decay when not considering indirect resistance. The green triangles represent the persister cells and their descendants. The orange squares represent the non-persister cells and their descendants. The burgundy star represents the experimental results from ref.[14]. The black cross represents the sum of green triangles with red squares at the end of the simulation. Because the error margin is not zero, the asterisk (experimental data) and the black cross (simulations) do not match in most cases. We assumed that the persister population decays according to an exponential and that persister cells leave the dormant state as soon as the medium becomes detoxified.
