# Supplementary material for: Bacterial persistence is essential for susceptible cell survival in indirect resistance, mainly for lower cell densities
Source: PLoS One. 2021 Sep 2;16(9):e0246500. doi: 10.1371/journal.pone.0246500 (PMC8412311; doi:10.1371/journal.pone.0246500)
Supplement: S4 Table — Results of simulations when we assumed that the persister population decays according to a power-law and that persister cells do not leave the dormant state as soon as the medium becomes detoxified. (DOCX) [file pone.0246500.s013.docx]

| S4 Table - Persister and non-persister cells that originated the final susceptible population considering τ_0_ = 60, $\boldsymbol{k}_{\boldsymbol{1}}$ = 0.07, $\boldsymbol{\beta}$ = -2.2* | | | | | |
| --- | --- | --- | --- | --- | --- |
| **Density** | **Frequency** | **Persister bacteria (%)** | **Total non-persister survivors (without considering duplications)** | **Total persister survivors (without considering duplications)** | **Total dormant cells at the end of the simulations** |
| Low | 1R:99S | 100 | 0 | 584 | 584 |
|  | 50R:50S | 100 | 0 | 28 | 28 |
|  | 99R:1S | 100 | 0 | 8 | 8 |
| High | 1R:99S | 37 | 1 | 9959 | 9957 |
|  | 50R:50S | 25 | 422 | 3055 | 2231 |
|  | 99R:1S | 11 | 90446 | 44180 | 8371 |

* We assumed that the persister population decays according to a power-law and that persister cells do not leave the dormant state as soon as the medium becomes detoxified
