# Supplementary material for: Bacterial persistence is essential for susceptible cell survival in indirect resistance, mainly for lower cell densities
Source: PLoS One. 2021 Sep 2;16(9):e0246500. doi: 10.1371/journal.pone.0246500 (PMC8412311; doi:10.1371/journal.pone.0246500)
Supplement: S6 Table — Results of simulations when we assumed that the persister population decays according to an exponential and that persister cells do not leave the dormant state as soon as the medium becomes detoxified. (DOCX) [file pone.0246500.s015.docx]

| S6 Table - Persister and non-persister cells that originated the final susceptible population considering τ_0_ = 30, $\boldsymbol{k}_{\boldsymbol{1}}$ = 0.095, $\boldsymbol{k}_{\boldsymbol{2}}$ = 0.01* | | | | | |
| --- | --- | --- | --- | --- | --- |
| **Density** | **Frequency** | **Persister bacteria (%)** | **Total non-persister survivors (without considering duplications)** | **Total persister survivors (without considering duplications)** | **Total dormant cells at the end of the simulations** |
| Low | 1R:99S | 100 | 0 | 108 | 108 |
|  | 50R:50S | 100 | 0 | 5 | 5 |
|  | 99R:1S | 100 | 0 | 6 | 6 |
| High | 1R:99S | 100 | 0 | 4896 | 4881 |
|  | 50R:50S | 100 | 0 | 4325 | 2735 |
|  | 99R:1S | 100 | 0 | 317337 | 39398 |

* We assumed that the persister population decays according to an exponential and that persister cells do not leave the dormant state as soon as the medium becomes detoxified
