# Supplementary material for: Bacterial persistence is essential for susceptible cell survival in indirect resistance, mainly for lower cell densities
Source: PLoS One. 2021 Sep 2;16(9):e0246500. doi: 10.1371/journal.pone.0246500 (PMC8412311; doi:10.1371/journal.pone.0246500)
Supplement: S10 Table — The exponent of a putative power-law describing the decay of the persister population. (DOCX) [file pone.0246500.s019.docx]

**S10 Table. Slope of the decay of the persister population assuming a power-law.**

| Reference | Figure in reference | Ampicillin concentration  (µg/mL) | β | R^2^ |
| --- | --- | --- | --- | --- |
| Moyed and Bertrand (1983) | 2a hipA+ | 100 | -4.65 | 0.98 |
| Moyed and Bertrand (1983) | 2a hipA7 | 100 | -0.72 | 0.74 |
| Balaban et al (2004) | 1a wt | 100 | -3.14 | 0.99 |
| Balaban et al (2004) | 1a hipA7 | 100 | -3.11 | 0.92 |
| Wiuff et al (2005) | 2 - Amp 24 µg/mL | 24 | -1.70 | 0.70 |
| Wiuff et al (2005) | 2 - Amp 128 µg/mL | 128 | -2.45 | 0.81 |
| Levin and Rozen (2006) | 1a | 128 | -4.07 | 0.97 |
| Orman and Brynildsen (2015) | 2 - KCN 22 h | 200 | -1.12 | 1.00 |
| Orman and Brynildsen (2015) | 2 - untreated | 200 | -1.85 | 0.98 |
| Orman and Brynildsen (2015) | 2 - KCN 6 h | 200 | -2.25 | 0.95 |
| Orman and Brynildsen (2015) | 2 - anaerobic 22 h | 200 | -1.87 | 0.96 |
| Orman and Brynildsen (2015) | 2 - aerobic | 200 | -1.91 | 0.97 |
| Orman and Brynildsen (2015) | 2 - anaerobic 6 h | 200 | -2.34 | 0.90 |
|  | | | |  |
